# Coming to terms with the nonmedical use of prescription medications

**DOI:** 10.1186/1747-597X-3-22

**Published:** 2008-11-18

**Authors:** Carol J Boyd, Sean E McCabe

**Affiliations:** 1 Institute for Research on Women and Gender, Substance Abuse Research Center and Nursing and Women's Studies, the University of Michigan, 204 S. State Street, Ann Arbor, Michigan 48109-1290, USA; 2 Substance Abuse Research Center and Institute for Research on Women and Gender, the University of Michigan, 2025 Traverwood Drive, Suite C, Ann Arbor, Michigan 48105-2194, USA

## Abstract

In this commentary we highlight limitations with the way nonmedical use of prescription medications has been measured in U.S. national studies. We also offer an alternative way of conceptualizing the nonmedical use of prescription medications for future study.

## 

The term *nonmedical use of prescription medications *is often used by researchers to describe the misuse of controlled medications by people who misuse someone else's prescription medication(s) or patients who misuse their own prescription medications (without a physician's knowledge). Nonmedical users may abuse their own medicines via non-oral routes to get high (e.g., snorting) while other nonmedical users may be given pills (for instance, from a family member) to self-treat a medical condition. Those with their own prescriptions may divert their medications to nonmedical users, while some nonmedical users may engage in doctor shopping in order to obtain a "legal" prescription. Thus, the term *nonmedical use of prescription medications *has come to characterize a heterogeneous group of motivations and related behaviors.

Despite the recent attention devoted to this form of drug abuse, relatively little is known about the different behaviors associated with the nonmedical use of prescription medications. Neither misuse of one's own medications nor motivations to engage in nonmedical use are adequately assessed in many of the existing epidemiologic studies that focus on substance abuse, including the *National Survey on Drug Use and Health *(NSDUH) [1], *Monitoring the Future *(MTF) [2], and the *National Epidemiologic Survey on Alcohol and Related Conditions (NESARC) *[3]. While these large studies have contributed to our initial understanding of nonmedical use, it is equally true that they have failed to advance our understanding of the diversity of behaviors associated with the nonmedical use of prescription medications.

Each of the three national studies relies on somewhat different survey questions to assess the nonmedical use of prescription medications – and each has limitations. *Monitoring the Future *uses the most straightforward question, stipulating that the drug *was taken without a doctor's order*, although whether respondents are using their own prescription medications or using diverted medications is not determinable. The NSDUH and the NESARC both use one complex question, stipulating that the drug was *not prescribed and then each adding, "was taken for the experience or feeling it caused" (NSDUH) or "to feel more alert, to relax or quiet nerves, to feel better, to enjoy themselves, or to get high or just to see how they would work" (NESARC)*. The NESARC question also stipulates an increase in amount, frequency, and/or longer duration than prescribed. A complex question means that respondents may answer affirmatively to the question but only meet one of the stipulated conditions, making it impossible for researchers to determine exactly which behaviors were endorsed.

Fifteen years ago, Hubbard and colleagues [4] critiqued the way in which "nonmedical use of prescription drugs" was often measured. In their paper, they noted that the NSDUH question used to measure nonmedical use was problematic because respondents were expected to recall multiple sets of information in one question. In the current NSDUH, respondents must decide: a) whether they used a controlled drug without a prescription, or b) whether they used a controlled medication for the experience or feeling it caused, or c) whether they used the controlled medication for some other reason. The NESARC question builds on the NSDUH but then adds even more information to the question. That is, respondents must decide if the controlled medication was: a) taken in greater amounts, or b) more often, or c) longer than prescribed.

There are other problems with the questions about nonmedical use in the national surveys. In one nonmedical use question, the NSDUH combines illicitly produced or "street" versions of drugs with controlled prescription medications. For instance, prescription stimulants such as methamphetamine, Desoxyn^®^, "uppers," "speed," Ritalin^® ^or methylphenidate are all combined within one question. Thus, researchers using the NSDUH stimulant data are forced to use the term "prescription-type" medications when writing about the nonmedical use of stimulant medications. *Monitoring the Future *and the NESARC have similar problems. In the MTF, the same question asks about "street" amphetamines (e.g., crystal methamphetamine) and prescription medications (e.g. Ritalin^®^), although some versions of the MTF specifically ask about individual prescription amphetamines. This forces researchers using the MTF data to use the term "prescription-type" medications when providing prevalence estimates for nonmedical use of amphetamines because street amphetamines are included in the question. The NESARC (Wave 2) has a somewhat different problem. It combines opioid analgesics with Cox-2 inhibitors in a single question about the nonmedical use of prescription pain medications. Thus, a respondent may be categorized as a nonmedical user of prescription pain medications BUT may never have used a prescription opioid (either medically or nonmedically). A Cox-2 inhibitor has no abuse potential and is not controlled (scheduled) medication, while all prescription opioid medications have abuse liability and are controlled. In the NESARC (Wave 2), these medications are "lumped" together into one question.

Given the limitations with the nonmedical questions in the national studies, it is virtually impossible to verify subtypes of nonmedical users from these studies, subtypes that have only been described in smaller, exploratory studies [5-8]. In particular, the national studies make it very difficult to determine whether the nonmedical user was: using his own controlled medication; using someone else's diverted medication; only using a controlled medication (and not a street drug), or using a controlled medication for the experience it caused or for some other reason.

The measurement limitations related to the "nonmedical use of prescription medications" obscures the complexity of this behavior and its related motives. It is for this reason that we propose that future researchers craft questions that disaggregate nonmedical use from motive and possession (see Table 1), thereby producing data that could better help researchers study the health consequences of the nonmedical use of controlled medications and assist prevention and treatment experts to craft more targeted messages.

**Table 1:** Subtypes of the nonmedical use of controlled drug classes

	Person **does not **possess a legal prescription	Person **does **possess a legal prescription
**MOTIVATED BY:**		
• Desire to get high; create an altered state or experimentation.	**Type 1:** **A problem behavior that is motivated by the desire to experiment, to get high, or to create an altered state with someone else's medication**.	**Type 3:** **A problem behavior that is motivated by the desire to experiment, to get high, or to create an altered state with one's own medication**.
RELATED BEHAVIORS:		
• Use in "recreational" settings		
• Use for reasons other than self-treatment		
• Use of scheduled medication with alcohol or other drugs simultaneously (co-ingestion)		
• May use alternate route of administration (IV, snorting)		

**MOTIVATED BY:**		
• Desire to alleviate symptoms of an actual or perceived health condition; uses medication for its pharmacological purpose (e.g., uses sleeping medication for insomnia) but does NOT involve mixing with alcohol or other drugs or non-therapeutic routes of administration.	**Type 2:** **A problem behavior motivated by the desire to self-treat with someone else's medication**.	**Type 4:** **A problem behavior motivated by the desire to self-treat with one's own medication but without the approval of the prescribing professional**.
RELATED BEHAVIORS:		
• May include an increase in the recommended frequency, dose, or duration		

## Competing interests

The authors declare that they have no competing interests.

## Authors' contributions

CJB and SEM conceived of the editorial, debated and worked through the conceptualization, and drafted the manuscript together. Both authors read and approved the final manuscript.
